# Prolonged-acting, Multi-targeting Gallium Nanoparticles Potently Inhibit Growth of Both HIV and Mycobacteria in Co-Infected Human Macrophages

**DOI:** 10.1038/srep08824

**Published:** 2015-03-06

**Authors:** Prabagaran Narayanasamy, Barbara L. Switzer, Bradley E. Britigan

**Affiliations:** 1 Department of Pathology and Microbiology, College of Medicine, University of Nebraska Medical Center, Omaha, 68198 Nebraska; 2 Department of Internal Medicine, College of Medicine, University of Nebraska Medical Center, Omaha, 68198 Nebraska; 3 Research Service, VA Medical Center- Nebraska Western Iowa, Omaha, 68105 Nebraska

## Abstract

Human immunodeficiency virus (HIV) infection and *Mycobacterium tuberculosis* (TB) are responsible for two of the major global human infectious diseases that result in significant morbidity, mortality and socioeconomic impact. Furthermore, severity and disease prevention of both infections is enhanced by co-infection. Parallel limitations also exist in access to effective drug therapy and the emergence of resistance. Furthermore, drug-drug interactions have proven problematic during treatment of co-incident HIV and TB infections. Thus, improvements in drug access and simplified treatment regimens are needed immediately. One of the key host cells infected by both HIV and TB is the mononuclear phagocyte (MP; monocyte, macrophage and dendritic cell). Therefore, we hypothesized that one way this can be achieved is through drug-targeting by a nanoformulated drug that ideally would be active against both HIV and TB. Accordingly, we validated macrophage targeted long acting (sustained drug release) gallium (Ga) nanoformulation against HIV-mycobacterium co-infection. The multi-targeted Ga nanoparticle agent inhibited growth of both HIV and TB in the macrophage. The Ga nanoparticles reduced the growth of mycobacterium and HIV for up to 15 days following single drug loading. These results provide a potential new approach to treat HIV-TB co-infection that could eventually lead to improved clinical outcomes.

The World Health Organization estimates that there are approximately 35 million people in the world infected with HIV and among that 1.8 million people die every year. Approximately one third of HIV infected individuals are co-infected with TB^1^,^2^. In addition, TB infection of HIV-1 positive patients appears to enhance HIV-1 replication, resulting in increased HIV-1 viremia, and hastens the progression of HIV-1 disease. Furthermore, HIV-1 infection in itself may impair appropriate immune response to TB, enhancing the progression and severity of TB. In this context, the design and development of long-acting formulations of traditional anti-TB and anti-HIV drugs has been of great interest. One limitation to the ability to simultaneously treat HIV and TB infection has been the drug-drug interactions of many standard anti-HIV and anti-TB drugs.

Mononuclear phagocytes (MP) are reservoirs for both HIV-1 virus and TB. In HIV infected human monocyte-derived macrophages (MDM), TNF-α was unable to exert its physiological anti-mycobacterial activity^3^. Given that simultaneous inhibition of HIV and TB replication could enhance the host response and control of these infections, we have worked to develop MP-targeted nanoformulations^4^,^5^ of anti-HIV-TB drugs using human monocyte-derived macrophages (MDM) and mycobacteria-lentivirus-macrophage interactions^6^,^7^ as part of an established TB drug discovery research program^1^,^8^,^9^,^10^,^11^,^12^,^13^,^14^,^15^.

Recently, there have been many developments in the long-acting targeted nanomedicines for HIV and bacterial infection separately, including long-acting anti-retroviral therapy nanoparticles (nanoART)^15^,^16^,^17^,^18^. Treatment of HIV-TB co-infection should also address the challenge of the significant pharmacokinetic drug-drug interactions between TB drugs and HIV drugs. Therefore, the designed nanoparticles should ideally be delivered as single or combination therapy, bypassing drug-drug interaction *via* a novel agent.

Iron (Fe) is crucial to the metabolism and growth of most microbes, including *M.tb* and HIV. Several important *M.tb* enzymes that are vital for its survival in human phagocytes require Fe. Among those are: superoxide dismutase and catalases that protect *M.tb* from phagocyte-derived reactive oxygen species (ROS); ribonucleotide reductase, which catalyzes the first step in DNA synthesis; and Fe-containing cytochromes/enzymes needed for oxidative phosphorylation. In addition, many of the *M.tb* genes are regulated by Fe via the Fe repressor protein, IdeR. Thus, alterations in Fe will affect many aspects of *M.tb* metabolism not directly tied to Fe utilization^19^,^20^,^21^,^22^.

Gallium (Ga) is a metal with many similarities to iron. Unlike Fe^+3^, Ga^+3^ cannot be reduced, and thus once bound to Fe binding sites in an enzyme protein, the enzyme is rendered inactive. Furthermore, many Fe binding proteins, such as bacterial siderophores, are unable to distinguish Ga^+3^ from Fe^+3^. Thus, all Fe dependent pathways in bacteria and virus would be potentially disrupted by the presence of Ga, leading to growth inhibition and killing. The Fe dependency of bacteria and virus for growth and pathogenicity suggests that selective pressures to reduce Ga acquisition would also result in poor Fe uptake, a counterproductive mutational change from the standpoint of bacterial and viral vitality. Ga is also not susceptible to classical drug efflux pumps and therefore, Ga should be less vulnerable to generally encountered antibiotic resistance mechanisms.

Gallium, in the form of nitrate, is a FDA-approved drug for the treatment of hypercalcemia of malignancy. Over a decade ago we were the first to propose that Ga could serve as therapy against human infections^10^,^11^,^23^,^24^. Subsequent work has demonstrated Ga-based therapies to be effective against a variety of bacterial pathogens, both *in vitro* and in murine models. Mycobacterial infections have been among those shown to be responsive to Ga treatment. To increase the long lasting presence of Ga in the host cell, we sought to synthesize Ga nanoparticles. Such nanoparticles should stay inside the macrophages for a longer time because of their physical nature. In addition, the multi-targeting Ga loaded macrophages could easily deliver drugs to the reservoirs, to eradicate disease.

## Results and Discussion

**Figure 1:**
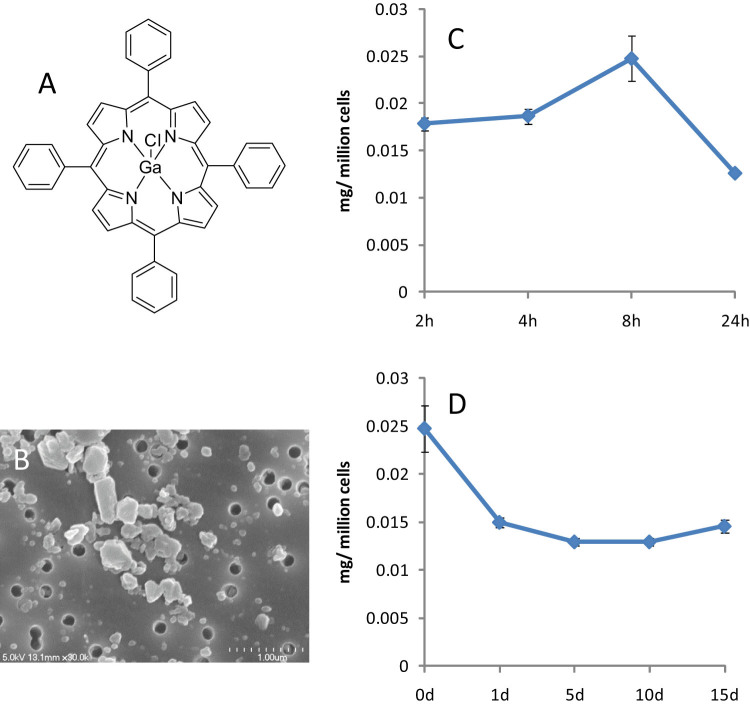
Properties of Ga-NP. (A). Chemical structure of Ga tetraphenyl porphyrin. (B). SEM picture of Ga-NP. (C). Uptake of Ga in macrophage; there is a maximum uptake at 8 h, up to 25 μg per million cells. (D). Retention of Ga in macrophage was studied up to 15 days. Retention of Ga for up to 15 days was observed along with sustained release of Ga particles. Data are shown as mean +/− s.e.m. for n = 3 replicate wells.

Ga tetraphenyl porphyrin (Ga-TP) (Fig. 1A) nanoparticle (Ga-NP) was synthesized by homogenizer using p407^25^ polymer. The synthesized nanoparticle showed 305 nm of average size and 0.29 polydispersity. The +35 Z-potential was observed through DLS measurement. The nanoparticle contained 48% of Ga-TP, which is excellent drug loading. The SEM picture showed a rod like structure of the nanoparticle, (Fig. 1B) capable of entering and staying in macrophage. The uptake of Ga in macrophage was studied by loading 300 μM Ga-NP into MDM and following levels over time. Interestingly, at 8 h the drug uptake was 25 μg at maximum (Fig. 1C). The sustained release and prolonged acting ability of the Ga-NP was evaluated by following the drug loaded macrophage for 15 days. The results confirmed that there is a sustained release of Ga over that time period (Fig. 1D). This assured the possibility of prolonged action of the nanoparticles. No macrophage cytotoxicity due to the Ga-NP was observed over 24 h, by 3-(4,5-dimethylthiazol-2-yl)-2,5-diphenyltetrazolium bromide (MTT) assay. Gallium at concentrations up to 2 mM did not show cytotoxicity in the macrophage for up to 37 days^23^. CC_50_ for both Ga-TP and Ga-NP was > 2 mM.

**Figure 2:**
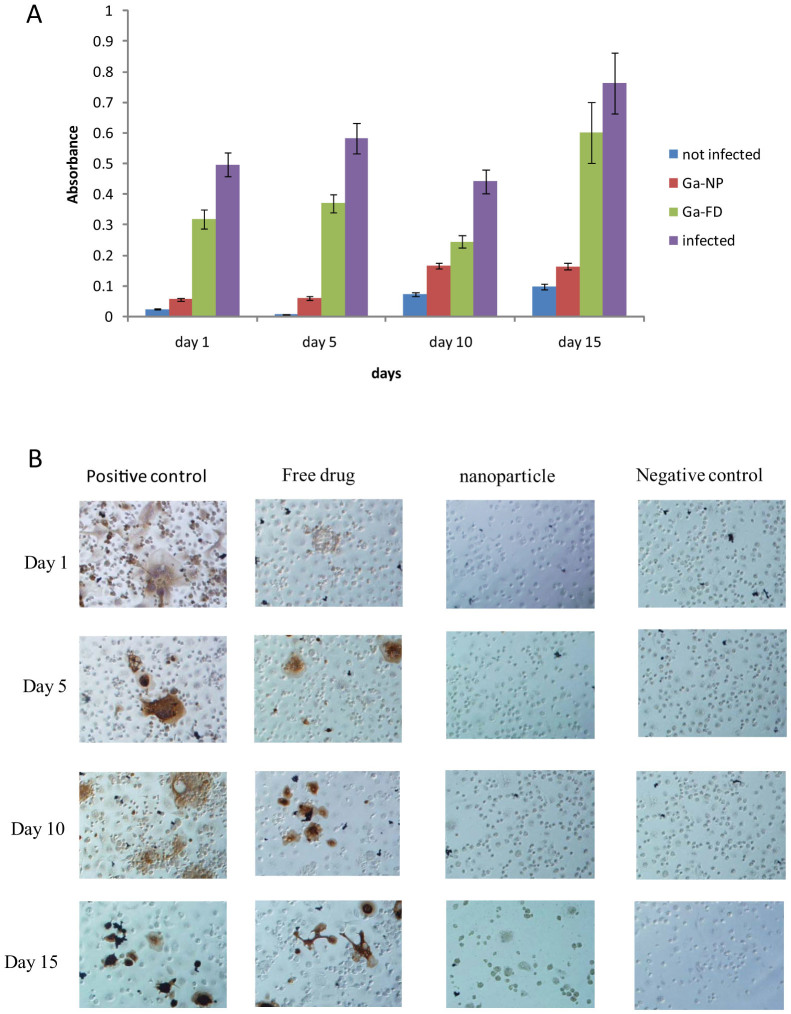
Activity of Ga-NP against individual pathogens. (A). Ga-NP activity against *M. smegmatis* was tested over time. Ga-NP inhibited the growth of *M. smegmatis*. It reduced the growth by more than 75% on 15^th^ day. In addition, it is more efficient than free Ga particles at day 15. Data are analysed using the t-test. Data are shown as mean +/− s.e.m. for n = 6, *P* < 0.05. (B). Prolonged activity of Ga-NP against HIV was tested by p24 staining. Ga-NP was effective up to 15 days.

Even though we have previously demonstrated broad anti-mycobacterial activity of Ga, the newly synthesized Ga-NP was examined for its long-lasting activity against *M. smegmatis* and compared with free drug (Ga-TP). The Ga-NP showed prolonged activity against *M. smegmatis* for 15 days, still reducing the growth four-fold on the 15^th^ day. In comparison, the free drug (i.e. non-nanoparticle Ga-TP) was not effective on day 15. Growth was closer to the infected positive control (Fig. 2A). These results encouraged us to explore the application of Ga-NP to other diseases.

Similar to bacteria, HIV infection is also associated with changes in iron metabolism, and an iron-mediated oxidative stress is likely to contribute to viral cytopathogenicity. Furthermore, it is interesting to point out that the interaction between iron and HIV may be reciprocal, since viruses with a life-cycle involving a DNA phase require chelatable iron for optimal replication. This combined evidence suggests that iron metabolism is an important area for virus/host interaction^26^.

There are at minimum five iron-dependent and iron regulated steps in HIV-1 replication: 1. The dNTPs generated by the iron-dependent protein ribonucleotide reductase are involved in viral RNA reverse transcription into DNA; 2. Iron can activate NF-kB by generating reactive oxygen species and activating IkB kinase, which leads to NF-kB localization to the nucleus; 3. At low cellular iron, CDK9 and cyclin T1 dissociate and Tat-mediated transcription is inhibited; 4. eIF5a, an iron dependent host protein involved in translation initiation, contains hypusine, which uses hypusine synthetase and deoxyhypusine hydroxylase; 5. Assembly of the Gag capsid proteins into mature virions requires the host cell protein ABCE1, an iron-binding ATPase^27^. All of these essential iron factors could be easily displaced by Ga through Ga-NP or competed with during protein synthesis. Preliminary studies reported in abstract form over a decade ago demonstrated that Ga was also able to inhibit replication of HIV-1 replication in the human U937 cell line. This suggested the possibility that Ga-based therapies could inhibit both mycobacteria and HIV-1 in co-infected cells.

To study the activity of Ga-NP against HIV, we loaded the Ga-NP into MDM for 8 h, followed by infecting the MDM with HIV at four different time periods (1, 5, 10, 15 days). p24 staining of MDM was then performed 12 days after HIV infection. We observed complete inhibition of HIV growth by Ga-NP when HIV infection occurred 1 and 5 days after GA-NP loading of the MDM. Inhibition of HIV-1 growth was still detectable for MDM infected with HIV 10 or 15 days post Ga-NP loading (Fig. 2B), although the effect was less than that observed with earlier infection time points. Thus, in addition to inhibiting mycobacterial replication in MDM, Ga-NP was able to inhibit HIV growth.

The observed results encouraged us to analyze the efficacy of Ga-NP against the HIV-mycobacterium co-infection and explore macrophages as drug delivery agents against co-infection. To analyze the mode of co-infection, we tested the Ga-NP under three different conditions. 1. Infection by HIV and bacteria simultaneously (Supplementary Fig. 1); 2. bacterial infection, followed by viral infection (Supplementary Fig. 2); and 3. viral infection, followed by bacterial infection (Supplementary Fig. 3). Interestingly, all infection protocols resulted in the same levels of mature virus and bacterial infection. However, when the viral infection occurred first, we observed more immature virus formation (Supplementary Fig. 2) but that did not create any effect in co-infection. Addition of anti-mycobacterial drugs that inhibited *M. smegmatis* growth had no effect on HIV growth (Supplementary Fig. 4). Similarly, an antiviral drug that blocked HIV replication did not reduce *M. smegmatis* growth (Supplementary Fig. 5). However, Ga-NP significantly inhibited both HIV and *M. smegmatis* growth (Supplementary Fig. 6). The results were comparable to that obtained with combination anti-retroviral/anti-mycobacterial therapy (nevirapine, tenofavir, rifampin and isoniazid). To our knowledge Ga is the first single drug demonstrating the capacity to successfully inhibit growth of both the virus and bacteria in HIV-mycobacterium co-infection.

**Figure 3:**
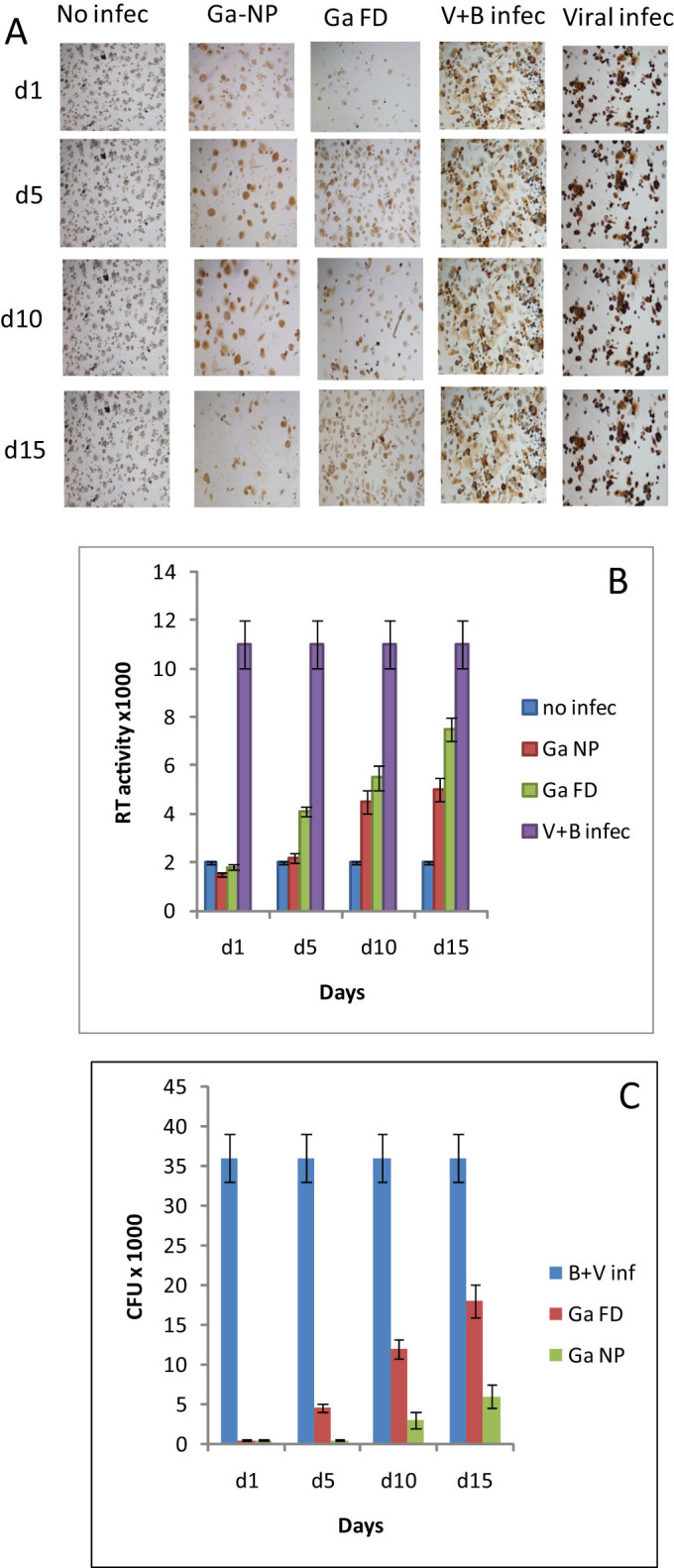
Activity of Ga-NP against co-infections. (A). Prolonged activity of Ga-NP against HIV-mycobacterium co-infection; intensity of HIV infection was tested by p24 staining. Ga-NP was able to reduce the HIV growth similar to the individual HIV infection up to 15 days. (B). Prolonged activity of Ga-NP against HIV-mycobacterium co-infection; intensity of HIV growth was tested by reverse transcriptase activity assay. There was less than 1% growth on days 1 and 5 for Ga-NP loaded macrophages. On day 15 around 25% growth of HIV was seen, better than free Ga particles. Data are analysed using the t-test. Data are shown as mean +/− s.e.m. for n = 9, *P* < 0.05. (C). Prolonged-acting property of Ga-NP against HIV-mycobacterium co-infection; mycobacterium growth was measured by CFU counting. Ga nanoparticle showed 7-fold less growth even after 15 days of single drug loading. Data are analysed using the t-test. Data are shown as mean +/− s.e.m. for n = 3, *P* < 0.05.

We next evaluated the duration of activity of the Ga-NP and free drug in HIV-*M. smegmatis* co-infected MDM. If infection occurred 1 day after drug loading of the MDM, no HIV or bacterial growth was detected. If infection was delayed until day 5 after drug loading, the bacteria and HIV started growing in free- drug loaded macrophages but not in Ga-NP loaded macrophages. Infection on day 15 post-drug loading yielded bacterial and HIV growth of up to 50% with free drug, whereas the Ga-NP loaded MDM showed less than 25% growth of HIV and bacteria when compared to positive control (Fig. 3).

**Figure 4:**
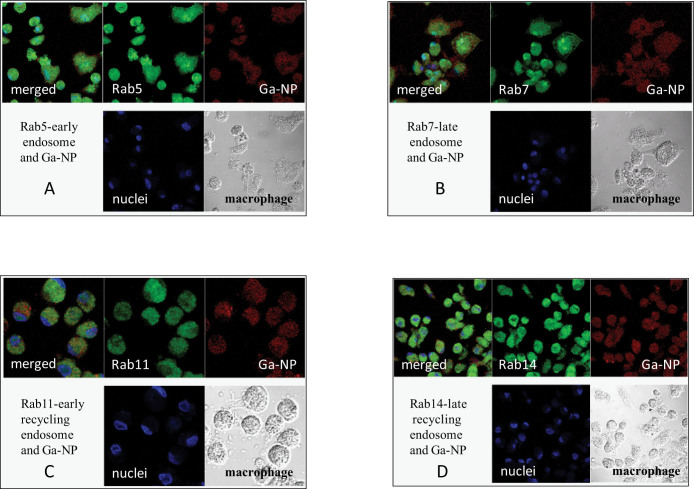
Confocal pictures of macrophages treated with fluorescent nanoparticle (red), nuclei (blue), macrophages (white), merged (all). Nanoparticles are seen in all sub cellular compartments confirming the multi targeted mechanism of gallium in macrophages. (A) Rab5 –early endosome, (B). Rab7- late endosome, (C). Rab11 – early recycling endosome, (D). Rab 14 – late recycling endosome. The control Ga-free nanoparticle showed very less nanoparticle in those compartments compared to Ga-NP.

To better understand the mechanism of Ga inhibition of HIV and mycobacterial replication, we carried out a subcellular trafficking study^15^. The subcellular distribution of Ga-NP was investigated to determine whether the nanoparticles undergo degradation and to determine the subcellular presence of Ga. A fluorescent tagged Ga-NP was synthesized and loaded into MDM. Since the mechanism of Rab5, Rab7, Rab11, Rab14 was well established in HIV studies^28^,^29^, we chose the same antibodies to evaluate our co-infection model. We observed the presence of nanoparticles in all the compartments, confirming the multi- targeting nature of the Ga against HIV and mycobacterium in each compartment (Fig. 4).

Prolonged-acting and multi-targeting Ga-NP was synthesized and successfully loaded into macrophages. The drug loading resulted in very high uptake, up to 25 μg/million cells. The drug loaded macrophage showed sustained drug release for up to 15 days. The Ga-TP and Ga-NP was effective in inhibiting the growth of *M. smegmatis* and HIV, when they infected the MDM separately. Importantly, Ga-TP and Ga-NP markedly inhibited the growth of both mycobacteria and HIV during MDM co-infection. The multi targeted prolonged-acting Ga-NP was effective up to 15 days after single drug loading. The subcellular trafficking of Ga-NP was determined and the presence of Ga-NP in all the compartments confirmed the multi-targeting approach. No drug to date has been shown to be active against both mycobacteria and HIV. The ability of Ga-TP and Ga-NP to control the growth of HIV, mycobacterium and co-infection could lead to a novel and effective approach to the treatment of HIV-TB that would also avoid the potential for drug-drug interactions.

## Methods

### Ethics Statement

We received the human samples from an already-existing collection and all samples were anonymized. Experiments with human samples are performed in full compliance with the National Institutes of Health. The methods were carried out in accordance with the approved University of Nebraska Medical Center ethical guidelines. All experimental protocols were approved by Institutional Review Board (IRB) # 162-93-FB with the title ‘Leukapheresis of normal donors for use in studies of disease pathogenesis and therapy'. We have the informed written consent from all participants involved in this study.

### Materials

Gallium (III) meso tetraphenylporphyrine chloride was purchased from Frontier Scientific (Logan, Utah, USA). Fluoresceinamine isomer 1; and Float-A-Lyser G2 were purchased from Sigma-Aldrich (St. Louis, MO, USA). Pooled human serum was purchased from Innovative Biologics (Herndon, VA, USA). Macrophage colony stimulating factor (MCSF) was prepared from 5/9m alpha3-18 cells (ATCC®, CRL-10154™) cultured in ATCC complete growth medium^30^. Rabbit anti-human antibodies to Rab 5, 7, 11 and 14, and Alexa Fluor 488 goat anti-rabbit IgG were purchased from Santa Cruz Biotechnology (Dallas, TX, USA).

### Preparation and characterization of the Ga nanoformulations

To 20 ml of 1% HEPES solution, 200 mg of Ga-TP, 100 mg of pluronic p407 polymer, 100 mg of sucrose was added and stirred overnight in dark conditions. The obtained mixture was homogenized until consistent size and polydispersity was attained. For preparation of suspensions by homogenization, mixtures were transferred to an Avestin C5 high-pressure homogenizer (Avestin Inc, Ottawa, ON) and extruded at 20,000 pounds per square inch until the consistent particle size was attained. Particle size, polydispersity (PDI), and surface charge (zeta potential) were determined by dynamic light scattering (DLS) using a Malvern Zetasizer Nano Series Nano-ZS (Malvern Instruments Inc, Westborough, MA). The resulting nanoparticle suspension was washed two times with 1% HEPES solution containing 0.5% p407 polymer through centrifugation (10000 rpm, 30 min at 4°C). The final pellet was resuspended in 1% HEPES and homogenized again to get the consistent polydispersity and size.

### Scanning electron microscopy

Scanning electron microscopy of the nanoparticles was carried out as described before^31^ using a Hitachi S4700 Field-Emission Scanning Electron Microscope (Hitachi High Technologies America Inc., Schaumburg, IL, USA) at UNL.

### Synthesis of fluorescent Ga nanoparticles

Dye labeled PLGA nanoparticles were prepared as previously described^32^. Briefly, PLGA was dissolved in DCM completely, followed by the addition N,N′-dicyclohexylcarbodiimide and N-hydroxysuccinimide (NHS) and stirred overnight at room temperature. The urea byproduct was filtered, the liquid was dried and the activated ester was used in the next step without further purification. Fluoresceinamine in DMSO was added to the activated NHS ester in DCM and stirred in the dark, and overnight at room temperature. DCM was then evaporated and the product precipitated using distilled water. The dye labeled polymer was purified by repeated dissolution in acetone and precipitation from ethanol, separated and then lyophilized. Dye-labeled PLGA was combined in a 1:3 ratio with non-labeled PLGA to manufacture dye-labeled nanoparticles.

PLGA nanoparticles containing Ga was prepared by double emulsification by sonication. Briefly, PLGA was dissolved in HPLC-grade dichloromethane (DCM). The drug was then added to the DCM/PLGA solution and mixed to obtain complete dissolution. This solution was added to 1% polyvinyl alcohol (PVA) cooled in an ice bath, and sonicated using a Cole Parmer Ultrasonic processor (Vernon Hills, IL, USA) at 20% amplitude for 10 min. Particle size, polydispersity index (PDI) and surface charge (zeta potential) were determined by dynamic light scattering (DLS) using a Malvern Zetasizer Nano Series Nano-ZS (Malvern Instruments Inc., Westborough, MA, USA). The suspension was mixed overnight at room temperature to evaporate DCM, then collected after 24 h or rotovaporised gently and centrifuged step-wise to 8000 × g at 5°C for 20 min. After decanting the supernatant, the pellet was washed twice in 25 mL of deionized water by centrifugation at 8000 × g for 20 min. The particle size was determined by DLS and drug concentrations were determined by ICP-MS^33^,^34^.

### Monocyte isolation and culture

Human monocytes were obtained by leukapheresis from HIV-1 and hepatitis B seronegative donors and purified by counter-current centrifugal elutriation^35^. Monocytes were cultured in Dulbecco's Modified Eagles Medium (DMEM) supplemented with 10% heat-inactivated human serum, 1% glutamine, 50 μg/mL gentamicin, 10 μg/mL ciprofloxacin, and 1000 U/mL MCSF at a cell density of 1 × 10^6^ cells/mL at 37°C in a 5% CO_2_ humidified atmosphere^36^. Seven days later, MDM were used for drug pharmacokinetics and anti-microbial assays.

MDM uptake and retention of nanoparticles and native drugs were determined as described^37^. Briefly, MDM were incubated with a range of drug concentrations, and cell uptake determined over a 24 h period. Adherent MDM were washed three times with phosphate buffered saline (PBS) and scraped into 1 mL PBS. Cells were pelleted by centrifugation at 1000 × g for 8 min at 4°C. The cell pellets were re-suspended in 200 μL of high performance liquid chromatography (HPLC)-grade methanol, sonicated, and centrifuged at 20,000 × g for 10 min at 4°C. The methanol extract was stored at −80°C until drug analysis. For cell drug retention studies, MDM were exposed to drug for 24 h, washed three times with PBS, and fresh DMEM without drug was added. MDM were cultured for an additional 15 days with half medium exchanges every other day. On days 1, 5, 10, and 15 following nanoparticle treatment, MDM were collected and methanol extracts prepared. The cell extract samples were stored at −80°C until drug analysis by Spectrometry with standard and co-efficient.

### Co-infection and Measurement of antimicrobial activity and antiviral activity

After drug treatment for 24 h, MDM were washed three times with PBS, then given fresh medium without drug. At days 1, 5, 10, and 15 following drug treatment, the MDM were infected with *M. smegmatis* (multiplicity of infection (MOI) = 1) and HIV-1_ADA_ (MOI = 0.01) in required order. Following 1 h of *M. smegmatis* infection (and 24 h of HIV-1 infection), cells were washed with PBS to remove extracellular mycobacterium and fresh medium was added. After 12 days, the cells were washed twice with PBS and scraped into 1 mL PBS. Both cell extract and media samples were stored at −80°C until analysis for mycobacterium and viral infection. Mycobacterium infection was determined by counting colony forming units (CFU) as previously described^15^,^23^ and viral infection was determined by P24 staining and RT activity as described below^38^.

### Measurement of RT activity

RT activity in cell medium samples were determined as previously described. Briefly, cell supernatant samples (10 μL) were mixed with 10 μL of 100 mM Tris-HCl (pH 7.9), 300 mM KCl, 10 mM DTT, and 0.1% nonyl phenoxylpolyethoxylethanol-40 (NP-40) in a 96-well plate. The samples were incubated at 37°C for 15 minutes. Twenty-five microliters of a solution containing 50 mM Tris-HCl (pH 7.9), 150 mM KCl, 5 mM DTT, 15 mM MgCl_2_, 0.05% NP-40, 10 μg/mL poly(A), 0.250 U/mL oligo d(T),12–18 and 10 μCi/mL^3^H-TTP was added to each well and incubated at 37°C for 18 h. Following incubation, 50 μL of ice-cold 10% trichloroacetic acid was added to each well, and the well contents harvested onto glass fiber filters and assessed for^3^H-TTP incorporation by β-scintillation spectroscopy^17^,^38^.

### HIV-1p24 antigen immunostaining

HIV-1p24 antigen staining was determined in PFA-fixed cells as previously described. Briefly, mouse monoclonal antibodies to HIV-1 p24 (1:100, Dako, Carpinteria, CA) were used to detect HIV-1 infected cells. Binding of p24 antibody was detected using Dako EnVision+ System-HRP labeled polymer antimouse secondary antibody and diaminobenzidine staining. Images were acquired using a Nikon TE300 microscope with a 20× objective (Nikon, Tokyo, Japan)^17^,^38^.

### Cytotoxicity

Cell viability was determined by the 3-(4,5-dimethylthiazol-2-yl)-2,5-diphenyltetrazolium bromide (MTT) assay as previously described^31^. Briefly, MDM were treated with either individual drugs or combinations at concentrations of 200, 300, or 400 μM for 24 h. The cells were washed with PBS and MTT solution (5 mg/mL) was added; the cells were incubated for 30 min at 37°C then washed with PBS. DMSO was added and incubated for 15 min at room temperature. Absorbance at 490 nm was quantitated using a SpectraMax M3 microplate reader (Molecular Devices, Sunnyvale, CA, USA).

### Measurement of antimicrobial activity

After drug treatment for 24 h, MDM were washed three times with PBS, then given fresh medium without drug. At days 1, 5, 10, and 15 following drug treatment, the MDM were infected with *M. smegmatis* (multiplicity of infection (MOI) = 1). Following 1 h of infection, cells were washed with PBS to remove extracellular mycobacterium and fresh medium was added. After 24 h, the cells were washed twice with PBS and scraped into 1 mL PBS and plated immediately. Mycobacterium infection was determined by counting colony forming units (CFU) as previously described^15^,^23^. Briefly, the cell suspension was treated with 0.25% SDS and diluted 100 times. The diluted samples were plated on 1.5% agar and incubated at 37°C for 3 days and the number of CFU were counted.

### Subcellular particle localization

For confocal imaging, monocytes were cultured on a 4-well Lab-Tek II CC2 chamber slide at a density of 0.5 × 10^6^ cells per well in the presence of 10% human serum and MCSF for 9 days. The cells were treated with 300 μM of fluorescein-labeled Ga nanoparticles for 8 h at 37°C, washed three times with PBS, fixed with 4% PFA for 30 min, permeabilized, and blocked with 0.1% Triton in PBS and 5% BSA in PBS and then quenched with 50 mM NH_4_Cl for 15 min. The cells were then washed with 0.1% Triton X-100 in PBS and incubated with (1:50) Rab 5, Rab 7, Rab 11 and Rab 14 primary antibodies for 1 h at 37°C. The cells were then washed and incubated with the secondary antibody conjugated to Alexa Fluor 488 for 45 minutes at 37°C. ProLong Gold anti-fade reagent with DAPI (Molecular Probes-Life Technologies, Grand Island, NY, USA) was added and slides were cover slipped and imaged with a Zeiss LSM 510 microscope (Carl Zeiss, Inc., Thornwood, NY, USA).

### Statistical analyses

Data analyses were carried out using Prism (GraphPad Software Inc, La Jolla, CA, USA). Significant differences in cytotoxicity response were determined by one-way ANOVA followed by Bonferroni's multiple comparisons test.

## Author Contributions

P.N. designed the experiments, executed the experiments, analyzed the results, and wrote the paper. B.S. purchased materials, analyzed results, and corrected the paper. B.B. suggested experiments, analyzed the results and corrected the paper.

## Supplementary Material

Supplementary Information Supplementary information
