# Author Correction: Prolonged-acting, Multi-targeting Gallium Nanoparticles Potently Inhibit Growth of Both HIV and Mycobacteria in Co-Infected Human Macrophages

**DOI:** 10.1038/s41598-021-97514-x

**Published:** 2021-09-01

**Authors:** Prabagaran Narayanasamy, Barbara L. Switzer, Bradley E. Britigan

**Affiliations:** 1 Department of Pathology and Microbiology, College of Medicine, University of Nebraska Medical Center, Omaha, NE 68198 USA; 2 Department of Internal Medicine, College of Medicine, University of Nebraska Medical Center, Omaha, NE 68198 USA; 3 Research Service, VA Medical Center- Nebraska Western Iowa, Omaha, NE 68105 USA

Correction to: *Scientific Reports* https://doi.org/10.1038/srep08824, published online 06 March 2015

We have become aware of errors in Figure 2 and Figure 3 of this Article resulting from the inadvertent interchange of some of the figure panels. We also believe that there is a need for clarification regarding the experimental design employed to generate the data reported in the Article and supplemental material. Two typographical errors in the text have also been identified. None of these errors alter the overall results or conclusions of the work as originally reported.

**Figure 2:**
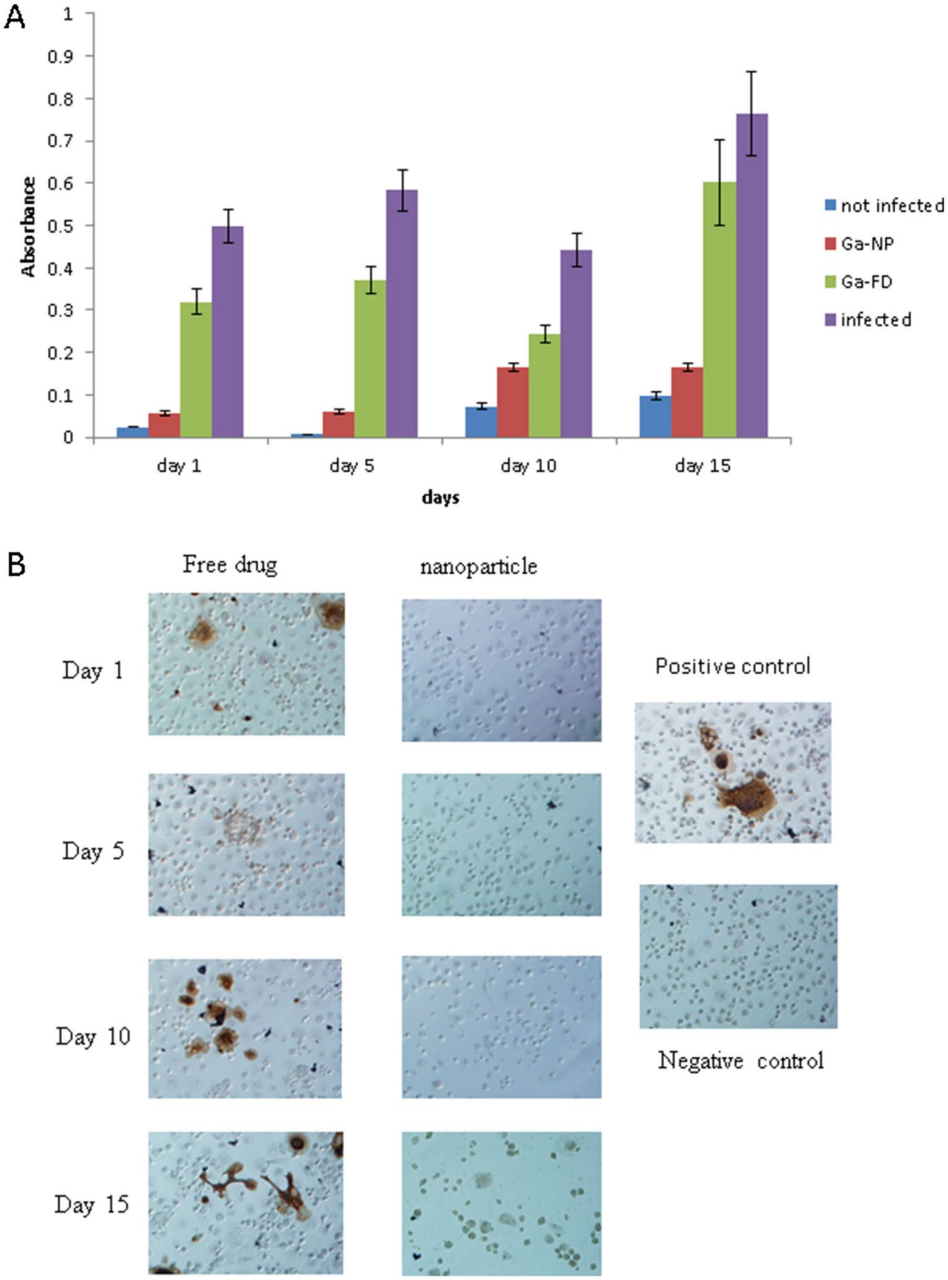
Activity of Ga-NP against individual pathogens. (**A**). Ga-NP activity against M. smegmatis was tested over time. Ga-NP inhibited the growth of M. smegmatis. It reduced the growth by more than 75% on 15th day. In addition, it is more efficient than free Ga particles at day 15. Data are analysed using the t-test. Data are shown as mean +/− s.e.m. for n = 6, P < 0.05. (**B**). Prolonged activity of Ga-NP against HIV was tested by p24 staining. Ga-NP was effective up to 15 days.

**Figure 3:**
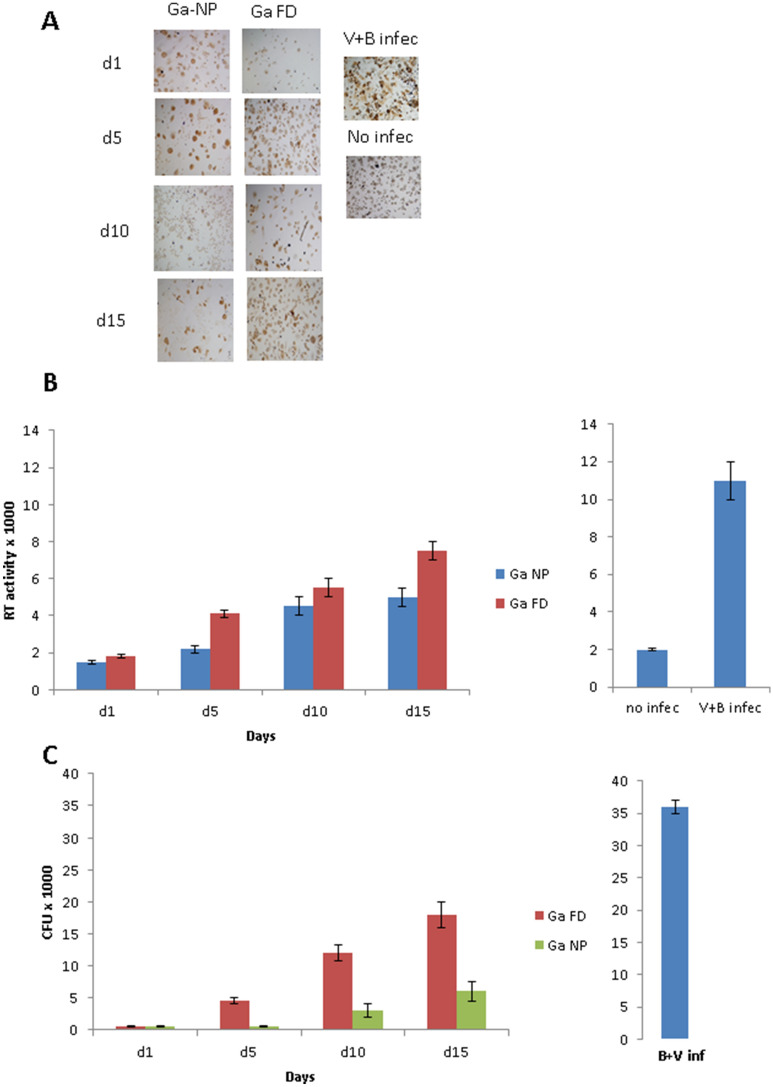
Activity of Ga-NP against co-infections. (**A**). Prolonged activity of Ga-NP against HIV-mycobacterium co-infection; intensity of HIV infection was tested by p24 staining. Ga-NP was able to reduce the HIV growth similar to the individual HIV infection up to 15 days. (**B**). Prolonged activity of Ga-NP against HIV-mycobacterium co-infection; intensity of HIV growth was tested by reverse transcriptase activity assay. There was less than 1% growth on days 1 and 5 for Ga-NP loaded macrophages. On day 15 around 25% growth of HIV was seen, better than free Ga particles. Data are analysed using the t-test. Data are shown as mean +/− s.e.m. for n = 9,* P* < 0.05. (**C**). Prolonged-acting property of Ga-NP against HIV-mycobacterium co-infection; mycobacterium growth was measured by CFU counting. Ga nanoparticle showed 7-fold less growth even after 15 days of single drug loading. Data are analysed using the t-test. Data are shown as mean +/− s.e.m. for n = 3,* P* < 0.05.

## The experimental design

The variable in each set of experiments reported in Figure 2 and Figure 3 was the number of days following loading with the nanoparticles prior to infection, not the duration of infection. The number of days in culture, and duration of infection (HIV or HIV/*M. smegmatis*), for each of the control samples for each of the different days post drug treatment was the same. Parallel cultures that served as positive (infection, no drug) and negative (no infection, no drug) controls for days 1, 5, 10, and 15 post-drug treatment infected samples were performed. As expected, all negative controls showed no infection and all positive controls showed high HIV or HIV/*M. smegmatis* growth. Rather than show different pictures of identical groups of cells, for the reader’s convenience and consistent with work of others,^1^ a single control picture and single control values are now used in Figures 2 and 3.

## Correction of errors for Figure 2

Four figure panels were accidently interchanged (cut/paste error) in Figure 2B: 1. free drug, day 1 with free drug, day 5; and 2. nanoparticle, day 10 with negative control, day 15. A copy/paste error of a panel (column 4/row 1) in Figure 2B was also detected and deleted. A corrected Figure 2 is now provided.

## Correction of errors/clarifications for Figure 3

The variable in the set of experiments summarized in Figure 3 was the days following loading with the nanoparticles prior to infection, not the duration of infection. All cell groups, except the negative control, were infected for the same time period. Rather than show different pictures of identical groups of cells, the same picture or value was used as the control in Figure 3A, 3B and 3C in the original Article. Since this could be confusing for some readers, a revised version of Figure 3 is provided as Figure 2 in this Correction, which contains separate and distinct controls in Figures 3A, 3B and 3C. A copy/paste error of a panel in Figure 3A, column 1/row 3 (Ga NP/day 10), was also detected. This has been corrected in the revised Figure.

## Clarification of experimental design for Supplementary Information

The experiments performed related to the Supplementary Information (SI Figure 1–4 and SI Figure 6) were carried out at the same time, i.e. in parallel. Therefore, the same control picture and values (positive, negative control) were used in all supplemental figures.

The Abstract states “The multi-targeted Ga nanoparticle agent inhibited growth of both HIV and TB in the macrophage.” *Mycobacterium tuberculosis* was not examined in this work, only *Mycobacterium smegmatis*. Thus, this section of the abstract should instead read “HIV and *Mycobacterium smegmatis*”.

In the Methods section: “Co-infection and Measurement of antimicrobial activity and antiviral activity”, “(MOI) = 1” should read “(MOI) = 0.1”. The Methods sections describing monocyte isolation and culture, cytotoxicity, measurement of antimicrobial activity and subcellular particle localization, were reproduced from the first author’s own article- reference 15 (FASEB J. 2014 Dec; 28(12): 5071–5082).

The entirety of this work and the errors necessitating the above corrections were extensively reviewed by panels of independent experts according to the policies and procedures of the University of Nebraska Medical Center regarding the possibility of scientific misconduct. No scientific misconduct was determined to have occurred.
