# Supplementary material for: Prolonged-acting, Multi-targeting Gallium Nanoparticles Potently Inhibit Growth of Both HIV and Mycobacteria in Co-Infected Human Macrophages
Source: Sci Rep. 2015 Mar 6;5:8824. doi: 10.1038/srep08824 (PMC4351534; doi:10.1038/srep08824)

Prolonged-acting, Multi-targeting Gallium Nanoparticles Potently Inhibit Growth of Both HIV and Mycobacteria in Co-Infected Human Macrophages

Prabagaran Narayanasamy[a*], Barbara L. Switzer[b,c] and Bradley E. Britigan[a,b,c*]

[a]Department of Pathology and Microbiology; [b]Department of Internal Medicine, College of Medicine, University of Nebraska Medical Center, Omaha, Nebraska, 68198 and Research Service, VA Medical Center- Nebraska Western Iowa, Omaha, Nebraska 68105

Corresponding authors: for PN: p.narayanasamy@unmc.edu; for BB: bradley.britigan@unmc.edu

Supporting information

Supplementary Figure 1: **Combined HIV-smegmatis infection and use of isoniazid, rifampin, tenofavir, nevirapine combination**. A). RT assay of HIV from co-infection showing no HIV growth, Data are analysed using the t-test. Data are shown as mean +/- s.e.m. for n = 9, $P < 0.05$. B). P24 staining of co-infected macrophage showing no HIV growth, C). CFU counting of mycobacteria from co-infection showing no bacterial growth. Data are analysed using the t-test. Data are shown as mean +/- s.e.m. for n = 3, $P < 0.05$.

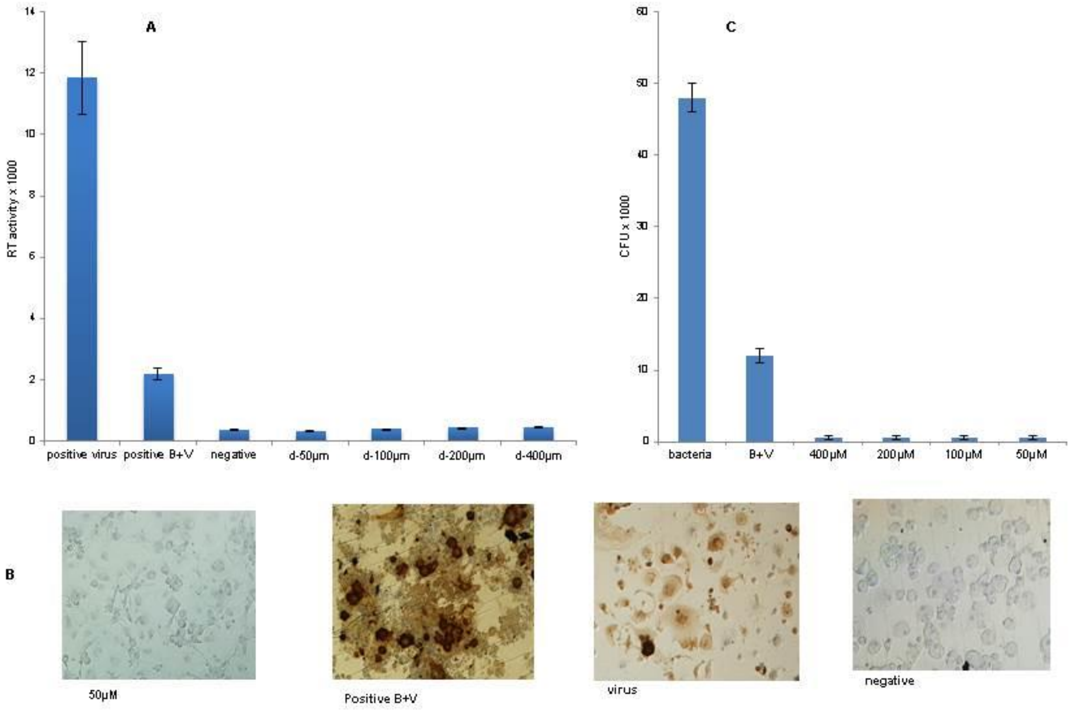

Supplementary Figure 2: **Bacterial infection followed by HIV infection and use of isoniazid, rifampin, tenofavir, nevirapine combination**. A). RT assay of HIV from co-infection showing no HIV growth, Data are analysed using the t-test. Data are shown as mean +/- s.e.m. for n = 9, $P < 0.05$. B). P24 staining of co-infected macrophage showing no HIV growth, C). CFU counting of mycobacteria from co-infection showing no bacterial growth. Data are analysed using the t-test. Data are shown as mean +/- s.e.m. for n = 3, $P < 0.05$.

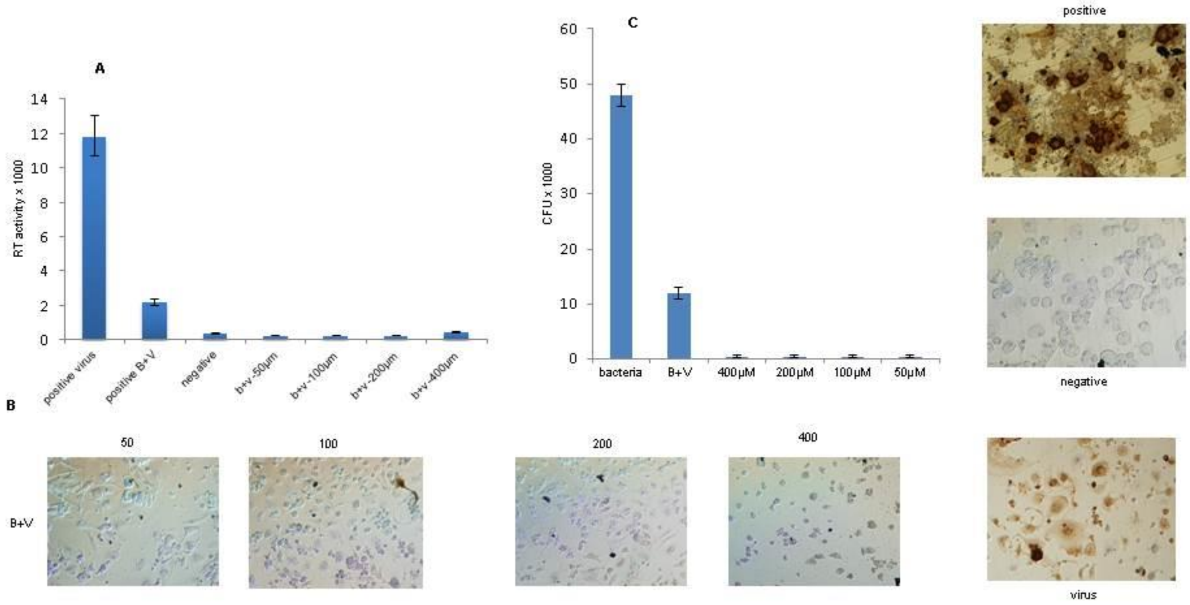

Supplementary Figure 3: **HIV infection followed by bacterial infection and use of isoniazid, rifampin, tenofavir, nevirapine combination.** A). RT assay of HIV from co-infection showing no HIV growth, Data are analysed using the t-test. Data are shown as mean +/- s.e.m. for n = 9, $P < 0.05$. B). P24 staining of co-infected macrophage showing little immature HIV growth, C). CFU counting of mycobacteria from co-infection showing no bacterial growth. Data are analysed using the t-test. Data are shown as mean +/- s.e.m. for n = 3, $P < 0.05$.

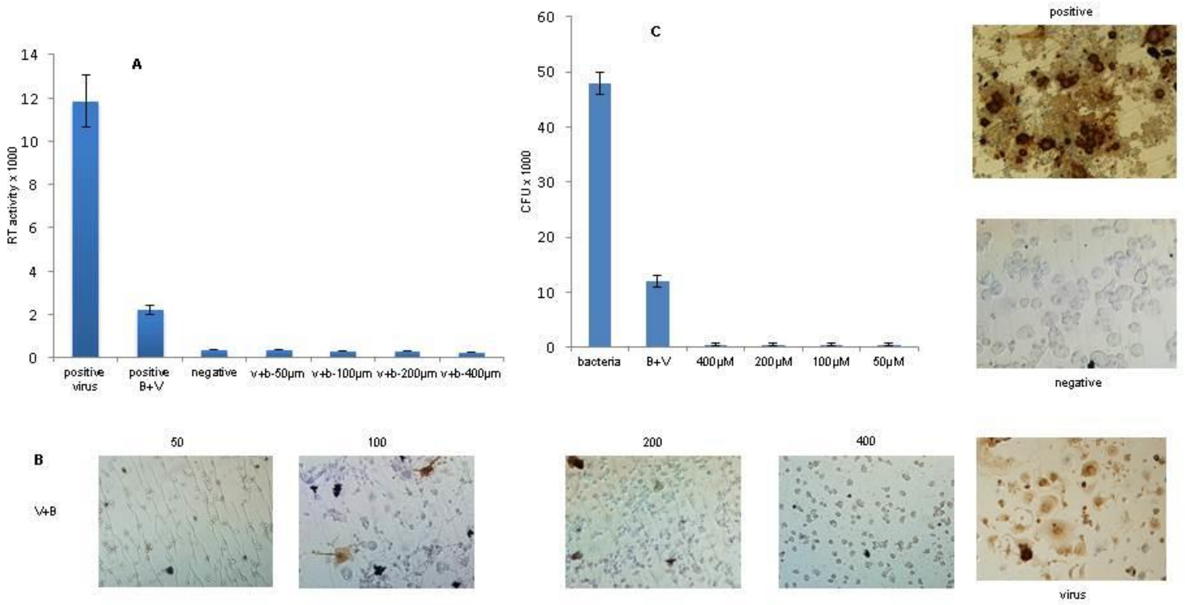

Supplementary Figure 4: **Combined HIV-smegmatis infection and use of anti-TB drug.** A). RT assay of HIV from co-infection showing normal HIV growth. Data are analysed using the t-test. Data are shown as mean +/- s.e.m. for n = 9, $P < 0.05$. B). P24 staining of co-infected macrophage showing normal HIV growth, C). CFU counting of mycobacteria from co-infection showing no bacterial growth. Data are analysed using the t-test. Data are shown as mean +/- s.e.m. for n = 3, $P < 0.05$.

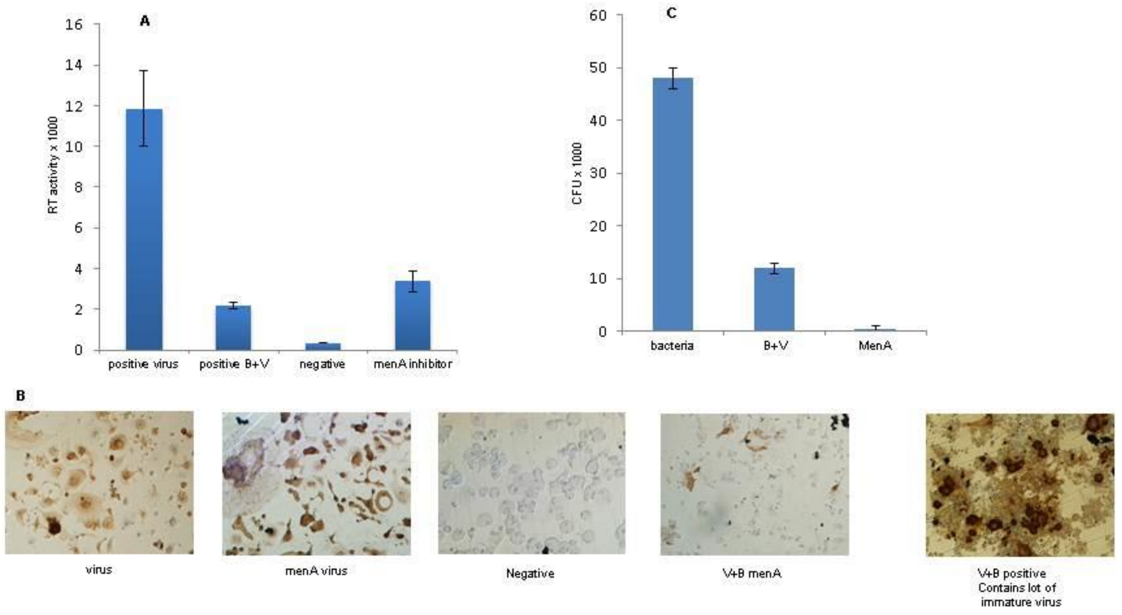

Supplementary Figure 5: **Combined HIV-smegmatis infection and use of antiviral drug (tenofavir).** A). RT assay of HIV from co-infection showing no HIV growth. Data are analysed using the t-test. Data are shown as mean +/- s.e.m. for n = 9, $P < 0.05$. B). P24 staining of co-infected macrophage showing no HIV growth, C). CFU counting of mycobacteria from co-infection showing normal bacterial growth. Data are analysed using the t-test. Data are shown as mean +/- s.e.m. for n = 3, $P < 0.05$

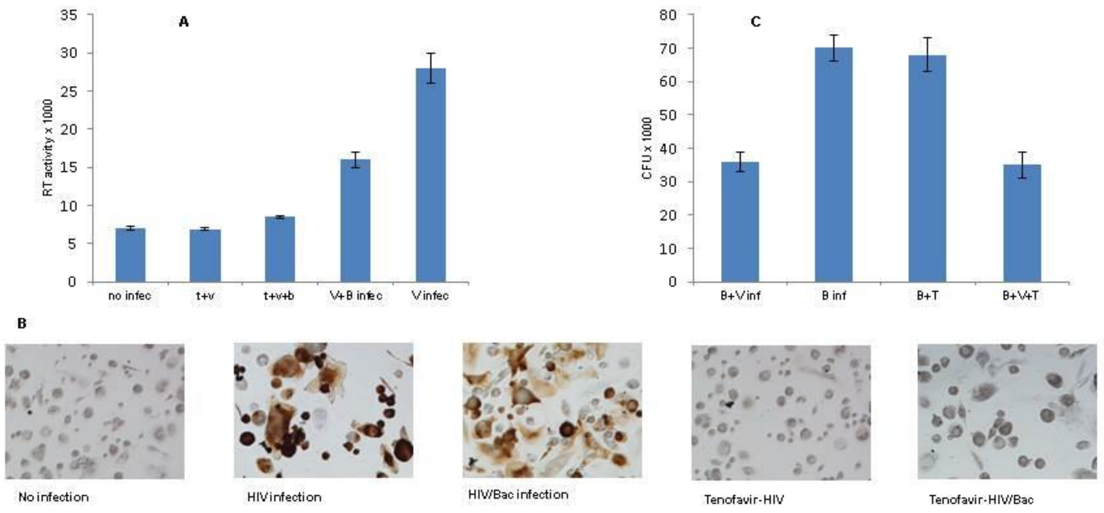

Supplementary Figure 6: **Combined HIV-smegmatis infection and use Ga-NP.** A). RT assay of HIV from co-infection showing no HIV growth. Data are analysed using the t-test. Data are shown as mean +/- s.e.m. for n = 9, $P < 0.05$. B). P24 staining of co-infected macrophage showing no HIV growth, C). CFU counting of mycobacteria from co-infection showing no bacterial growth. Data are analysed using the t-test. Data are shown as mean +/- s.e.m. for n = 3, $P < 0.05$.

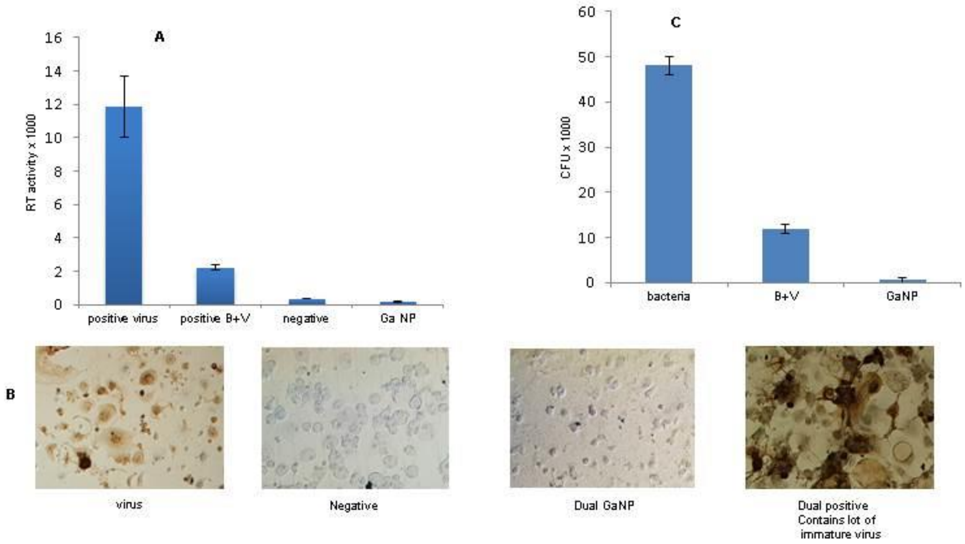

Supplement: Supplementary Information [file srep08824-s1.pdf]
